# GENERA: A Combined Genetic/Deep-Learning Algorithm for Multiobjective Target-Oriented De Novo Design

**DOI:** 10.1021/acs.jcim.3c00963

**Published:** 2023-08-09

**Authors:** Giuseppe Lamanna, Pietro Delre, Gilles Marcou, Michele Saviano, Alexandre Varnek, Dragos Horvath, Giuseppe Felice Mangiatordi

**Affiliations:** † Chemistry Department, University of Bari “Aldo Moro”, Via E. Orabona, 4, I-70125 Bari, Italy; ‡ CNR − Institute of Crystallography, Via Amendola 122/o, 70126 Bari, Italy; § Laboratoire de Chémoinformatique UMR7140, 4 rue Blaise Pascal, 67000 Strasbourg, France; ∥ CNR − Institute of Crystallography, Via Vivaldi 43, 81100 Caserta, Italy

## Abstract

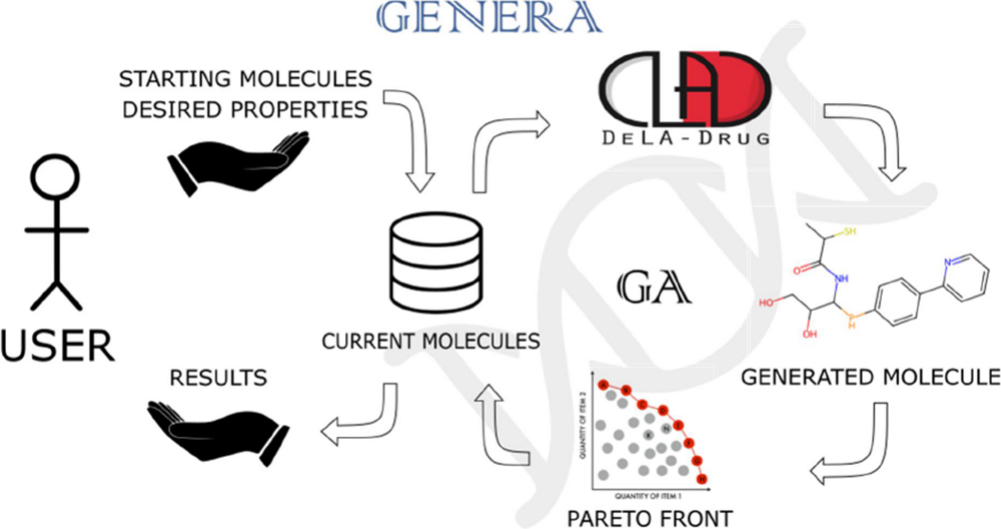

This study introduces a new de novo design algorithm called *GENERA* that combines the capabilities of a deep-learning algorithm for automated drug-like analogue design, called *DeLA-Drug*, with a genetic algorithm for generating molecules with desired target-oriented properties. Specifically, *GENERA* was applied to the angiotensin-converting enzyme 2 (ACE2) target, which is implicated in many pathological conditions, including COVID-19. The ability of *GENERA* to de novo design promising candidates for a specific target was assessed using two docking programs, PLANTS and GLIDE. A fitness function based on the Pareto dominance resulting from computed PLANTS and GLIDE scores was applied to demonstrate the algorithm’s ability to perform multiobjective optimizations effectively. GENERA can quickly generate focused libraries that produce better scores compared to a starting set of known ACE-2 binders. This study is the first to utilize a DL-based algorithm designed for analogue generation as a mutational operator within a GA framework, representing an innovative approach to target-oriented de novo design.

## Introduction

Drug discovery (DD) is costly and time-consuming.^1^ On average, bringing a new drug to the market takes 10 years and approximately 2.7 billion dollars.^2^ Given these constraints, the modern pharmaceutical industry prioritizes using computational tools to minimize the number of candidates that must undergo costly preclinical and clinical testing, thus saving money and speeding up the process. In recent years, new structure- and ligand-based models have emerged to tackle this challenging task.^3−5^ These predictive models have enabled the application of virtual screening (VS) strategies to identify promising candidates from vast libraries of both presynthesized and easily synthesizable compounds.^6−9^ The progression of artificial intelligence, particularly in the area of deep learning (DL), has led to the development of new techniques that have been effectively implemented.^10−18^ A significant distinction from traditional VS procedures is the origin of the compounds under consideration. In the VS strategy, the molecules assessed in silico are known a priori, whereas generative models endeavor to design the compounds for subsequent evaluation (de novo design). Specifically, DL-based algorithms can apprehend the patterns in extensive datasets and replicate those patterns in new samples with exceptional efficacy. DL-based methodologies offer a significant advantage in the drug-design context because they can automatically generate novel chemical structures with desired properties, such as the predicted affinity toward a specific target of interest. The literature contains several successful examples of generative model applications.^19−21^ It is worth noting that various architectures can be employed, including (i) recurrent neural networks (RNNs) with long short-term memory (LSTM) cells, (ii) auto-encoders, (iii) generative adversarial networks, and (iv) reinforcement learning (for an extensive review on this topic, the interested reader is referred to the work by Sousa et al.^22^ or Schneider and Clark^23^). In a recent co-authored paper, a new DL algorithm named *DeLA-Drug*(24) was proposed for a data-driven generation of drug-like analogues. The model, trained using SMILES strings syntax from over 1 million compounds extracted from ChEMBL28 (ChEMBL-DB), generates drug-like molecules from a single query using a new approach called sampling with substitutions (SWS). Unlike other methods employed in de novo drug design, the algorithm does not involve a fine-tuning step to steer the generation phase, making it (i) applicable in low-data regimes, where an extensive dataset of compounds with known experimental data is not available for the target of interest and (ii) implementable in an easy-to-use web platform (http://www.ba.ic.cnr.it/softwareic/deladrug), requiring only the 2D structure or the SMILES string of a seed (departure) compound. However, the structural patterns learned by *DeLA-Drug* from the entire ChEMBL are general, enabling the tool to generate broadly diverse libraries for primary screening rather than specific target-focused libraries. To overcome this limitation, we combined *DeLA-Drug* with a genetic algorithm (GA) to direct the generation toward (predicted) target-directed compounds, where the compound propensity to bind the target was estimated—here—by docking scores (alternatively, any 2D or 3D QSAR model may as well be used instead). The resulting computational workflow, called *GENERA*, was applied to the de novo inhibitor design of angiotensin-converting enzyme 2 (ACE2), which plays a crucial role in various pathological conditions, including COVID-19. Notably*, GENERA*’s ability to generate promising candidates for a specific target was assessed using two software, namely, Protein-Ligand ANT System^25^ (PLANTS) and Grid-based ligand docking with energetics^26^ (GLIDE), returning multiple scoring criteria. A fitness function based on Pareto dominance resulting from the computed docking scores was applied, demonstrating GENERA’s ability to perform multiobjective optimizations effectively. By starting with a limited set of compounds (e.g., a group of molecules already proven to be active toward the target of interest), GENERA was able to swiftly generate new candidates that (i) are chemically valid, (ii) explore a new chemical space, and (iii) are highly promising for further in vitro studies as observed to dock even better than confirmed actives of the starting set. From a methodological perspective, this study represents the first attempt to utilize a DL-based algorithm designed for analogue generation as a mutational operator within a GA framework.

## Materials and Methods

### GENERA Architecture

**Figure 1:**
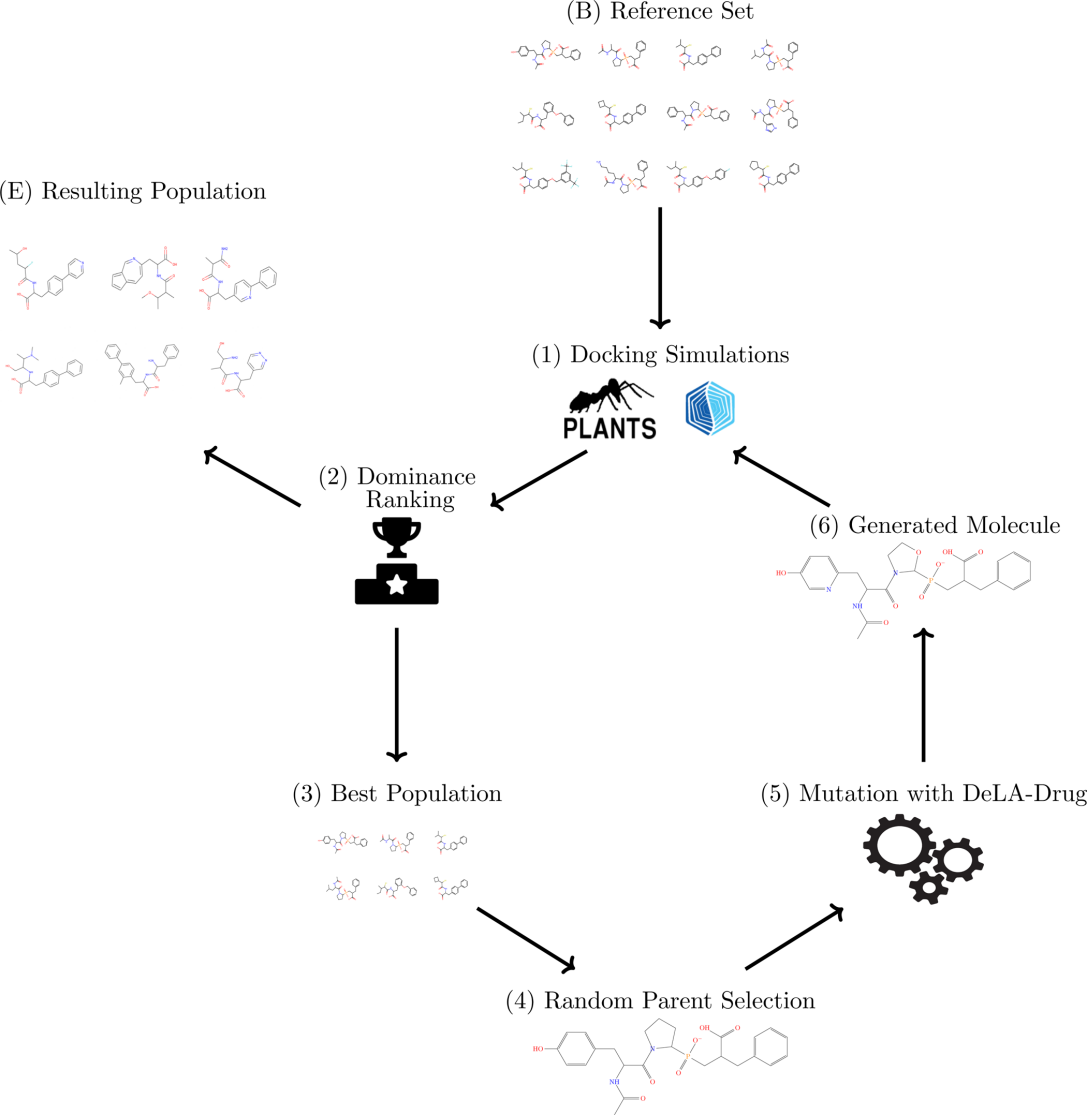
Flowchart showing the main steps of GENERA: (B) reference set of molecules. This step marks the beginning of the main loop constituting the algorithm; (1) evaluation of the dataset based on the chosen criteria (e.g., docking scores); (2) ranking based on Pareto dominance using selected objectives; (3) selection of a subpopulation of best-performing molecules based on the chosen fitness function; (4) random selection of one single parent in the best-performing molecules group; (5) application of the mutation operation to the parent using *DeLA-Drug*; (6) a child molecule is generated, checked for uniqueness, standardized, evaluated and added to the population so that the cycle can restart; (E) current population, this step is the possible exit point from the main loop constituting the algorithm.

In this work, we present GENERA, a novel algorithm that uses *DeLA-Drug*, a recently published generative model,^24^ as a mutational operator within a GA framework. Only molecules meeting specific criteria, such as producing promising docking scores, are eligible as new queries for subsequent generations (Figure 1). The algorithm can accommodate multiple fitness scores and potentially incorporate additional compound pertinence criteria from QSPR models, similarity scoring, and other factors. The following sections provide further details on *DeLA-Drug* and the GA methodology employed in this study.

### *DeLA-Drug*

As mentioned above, *DeLA-Drug* is a deep generative model that learns the syntax of the SMILES strings belonging to **1,092,285** compounds extracted from ChEMBL-28^27^ and generates drug-like analogues starting from a given query. The model is based on an RNN^28^ composed of two LSTM layers^29^ and generates compounds following an SWS approach whose basic concept consists in varying a user-defined number of characters—hereinafter referred to as substitutions (S)—of the starting SMILES string based on a conditional probability density function. The interested reader is referred to the published paper^24^ for all the details concerning *DeLA-Drug* architecture.

### Genetic Algorithm

The combination of *DeLA-Drug* with a GA leads to a new architecture whose main steps are displayed in Figure 1 and reported in the following:

A specific number of substitutions between 1 and 5 is randomly selected and applied to random positions of one of the available SMILES.
For each randomly selected parent compound, one child is generated. Notice that the validity of the generated structure is assessed by *DeLA-Drug*. If after 1000 attempts no valid structure is generated, the algorithm will proceed to select a new combination of substitution positions.
First, the child SMILES is standardized to its canonical form, then checked for uniqueness within the repository of already processed items, kept up to date by the GENERA script (vide infra). If the current SMILES has already been visited, the current attempt is abandoned. Otherwise, RDKit^30^ is used as the tool to identify reactive or chemically unstable groups by means of a series of fast substructure searches.
The candidate is discarded if such groups are found, setting its multiple fitness scores to very low levels.^31^ Otherwise, the algorithm proceeds to ligand preparation and docking into the binding site of interest, with both PLANTS^25^ and GLIDE,^26^ retrieving the corresponding fitness scores.
Based on the computed docking scores, nondominated compounds defining the Pareto front in the multiobjective space (Experimental Validation Status (EVS) and several docking-related properties, vide infra) are selected (best population) to be used as parents for a further generation.

### Reference Population and “Experimental Validation Status”

The “chromosome” of the GA is nothing but the SMILES string of the molecule. For this reason, the typical injunction to start a GA from a “random” initial population was not followed here. Albeit it is, in principle, feasible to start with any random pool of valid SMILES, we chose to select the already known experimentally validated ACE2 inhibitors as a starting point. The 42 most active ACE2 binders were extracted from ChEMBL^27^ release,^28^ standardized according to the default protocol of the Laboratory of Chemoinformatics,^32^ and their fitness scores were calculated by docking, using the same scripts used to estimate the fitness of new candidates to be generated by the GA. Thus, the evolutionary algorithm will be challenged to find analogues with docking scores better than state-of-the-art actives. Unfortunately, docking scores are only weakly correlated with affinity; therefore, some confirmed actives may not appear as “fit” according to the calculated docking scores, risking being eliminated from the Pareto front by newly generated inactive compounds with artefactually high docking scores. Therefore, an additional formal fitness score (EVS)—set to one for the ChEMBL-reported actives and zero for all the generated structures—was added as an additional objective, herewith ensuring that experimentally validated species will never be dominated by the generated ones and always remain within the population of eligible genitors. The SMILES of this reference population of actives, associated with their fitness scores as defined above, need to be installed in the GENERA working directory as the initial “best” pool of items eligible to have an offspring.

### Docking Simulations

All the chemicals belonging to the reference set*,* as well as those generated by *GENERA* using *DeLA-Drug*, were docked on the crystal structure of ACE2 in complex with the inhibitor XX5 (PDB code: 1R4L).^33^ The retrieved pdb file was prepared using the *Protein Preparation Wizard*,^34^ available from the Schrödinger suite 2022-4, to add all the missing hydrogens, assign appropriate charge states at physiological pH, and reconstruct incomplete side chains and rings. *LigPrep*,^35^ available from the Schrödinger suite 2022-4, was used to generate all the possible tautomers, stereoisomers, and ionization states at pH 7.0 ± 2.0. The obtained files were used for docking simulations using PLANTS^25^ and GLIDE^26^ programs. Full flexibility was allowed for the ligands during the docking process, while the protein was held fixed. PLANTS^25^ was used with default settings and the ChemPLP scoring function,^36^ with a cutoff radius of 12 Å around the binding site center (taken as the geometric center of the 1RL4 ligand). Ligands were submitted as issued by *LigPrep*(35) and passed to SPORES^37^ for PLANTS-compatible parameterization. From now on, we refer to the value of the PLANTS ChemPLP score^36^ (− ChemPLP score) only as plantsDS for readability. It was observed (unpublished results) that plantsDS tend to be strongly compound size dependent—there is a strong correlation between log(plantsDS) and the log of the number of heavy atoms *N*_h_, with a slope of 0.35. Therefore, selection by plantsDS alone tends to favor large species, whereas ligand efficiency plantsLE = plantsDS/*N*_h_ artificially favors small, fragment-like species. However, a pondered ligand efficiency score plantsPLE = plantsDS/*N*_h_^0.35^ will not depend on molecular size. When working with PLANTS^25^ data only, *GENERA* accounted for all these three criteria: plantsDS, plantsLE, and plantsPLE. Thus, more negative docking scores (e.g., higher plantsDS values) mean that “fitter” ligands entered as independent objectives accounted on the Pareto front.

The OPLS_2005^38^ force field, the standard precision (SP) protocol, and a grid centered on the cognate ligand with an edge of 10.00 Å for the inner box and 25.15 Å for the outer box were used for docking simulations performed by GLIDE.^26^ Additionally, during the grid generation, we set a metal coordination constraint on the zinc ion to adequately manage the presence of a metal in the ACE-2 binding site in the subsequent docking simulations. The GLIDE^26^ scoring function was used as a docking output, and a ligand efficiency (glideLE) was also considered. Notice that we refer to the negative value of the GLIDE score as glideDS, for readability. These protocols were tested by redocking the cognate ligand into the binding site. Both docking programs generated poses with the same binding mode as seen in the X-ray structure, being the computed root-mean-square deviation (RMSD) equal to 1.45 Å (GLIDE^26^) and 1.50 Å (PLANTS^25^). The obtained values support the robustness of the employed docking procedures.

### Pareto Multiobjective Optimization

Two multiobjectives optimizations were performed by using GENERA: (i) based on PLANTS^25^ only (using EVS, plantsDS, plantsLE, plantsPLE as objectives) and (ii) based on “consensus” docking by both PLANTS^25^ and GLIDE^26^ (using EVS, plantsDS, plantsLE, glideDS, glideLE) as objectives. GA was run asynchronously on a multinode, multicore Linux cluster. “Designer” scripts are started by the master GENERA script, using the locally installed scheduler/batch mechanism, on any free CPU (the user can specify a maximal number of cores to be claimed). These runs access (at their execution time) the current state of the file containing the “best” SMILES strings eligible to generate offspring, randomly extract one of the compounds, execute *DeLA-Drug*(24) to retrieve the “mutant” offspring, calculate the above-listed criteria by sequentially running PLANTS,^25^ and then eventually GLIDE^26^ on the allotted CPU core. Finally, the designer script writes a one-line text file with the offspring SMILES and its objective score values in the GENERA working directory, in which the master periodically checks for new entries of this type. When detected, these new entries are first concatenated to a repository of all so-far generated SMILES and their fitness scores. Then, the current “best” SMILES file and the new entries are merged and submitted to the Pareto front tool, which detects and deletes any “dominated” items at the input. An item is “dominated” if there exists at least one other item which is simultaneously better (strictly >) for all objectives. Technically, the dominance (*Dom*) of an item represents the number of dominating items in the set. The Pareto^39^ front, defined as the subset of nondominated items *Dom* = 0, is output by the Pareto front tool and then renamed to become the new “best” pool of items.

## Results and Discussion

### Generation Based on PLANTS Scores

**Figure 2:**
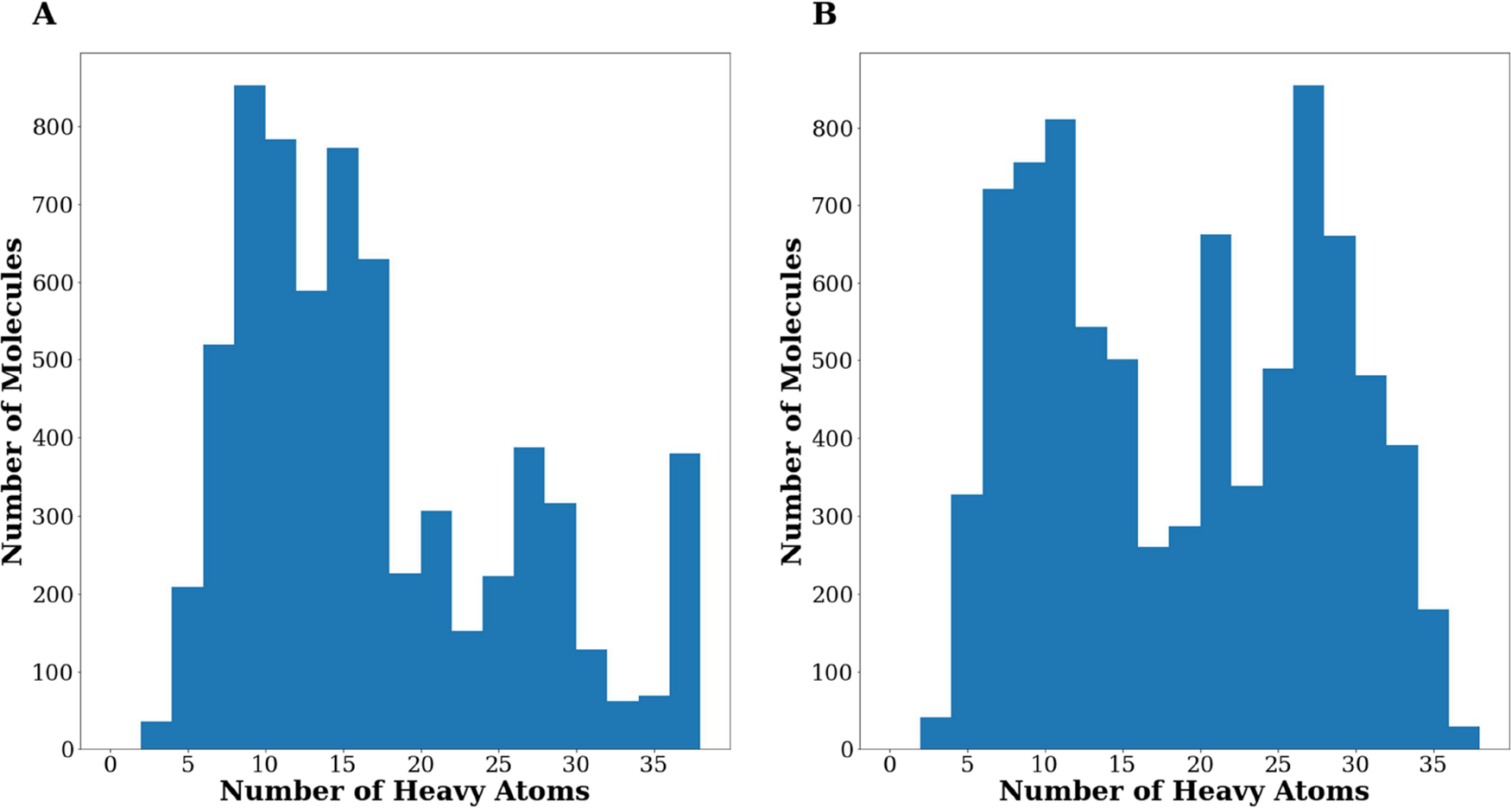
Distribution of the number of heavy atoms returned by the molecules belonging to (A) *Gen1* and (B) *Gen2* sets.

GENERA designed **6648** unique and chemically valid molecules (from now on *Gen1 set*) starting from the set of 42 initial active molecules targeting ACE-2 (reference set). As mentioned above, some objectives of the Pareto front depend on the size of the molecules. Specifically, the docking score favors larger molecules, while the ligand efficiency smaller ones.^40^Figure 2A displays a distribution plot of the heavy atoms in the *Gen1 set*. As evident in the Figure, GENERA can design molecules of various sizes, ranging from fragments with less than 5 heavy atoms to compounds with more than 35 heavy atoms. This behavior may be user-controlled: unless explicitly interested in fragment-based drug design or the design of building blocks for focused library synthesis, LE scores need not be included as objectives—they may be replaced by any specific constraints in terms of size, Lipinski rule compliance, and so forth.

**Figure 3:**
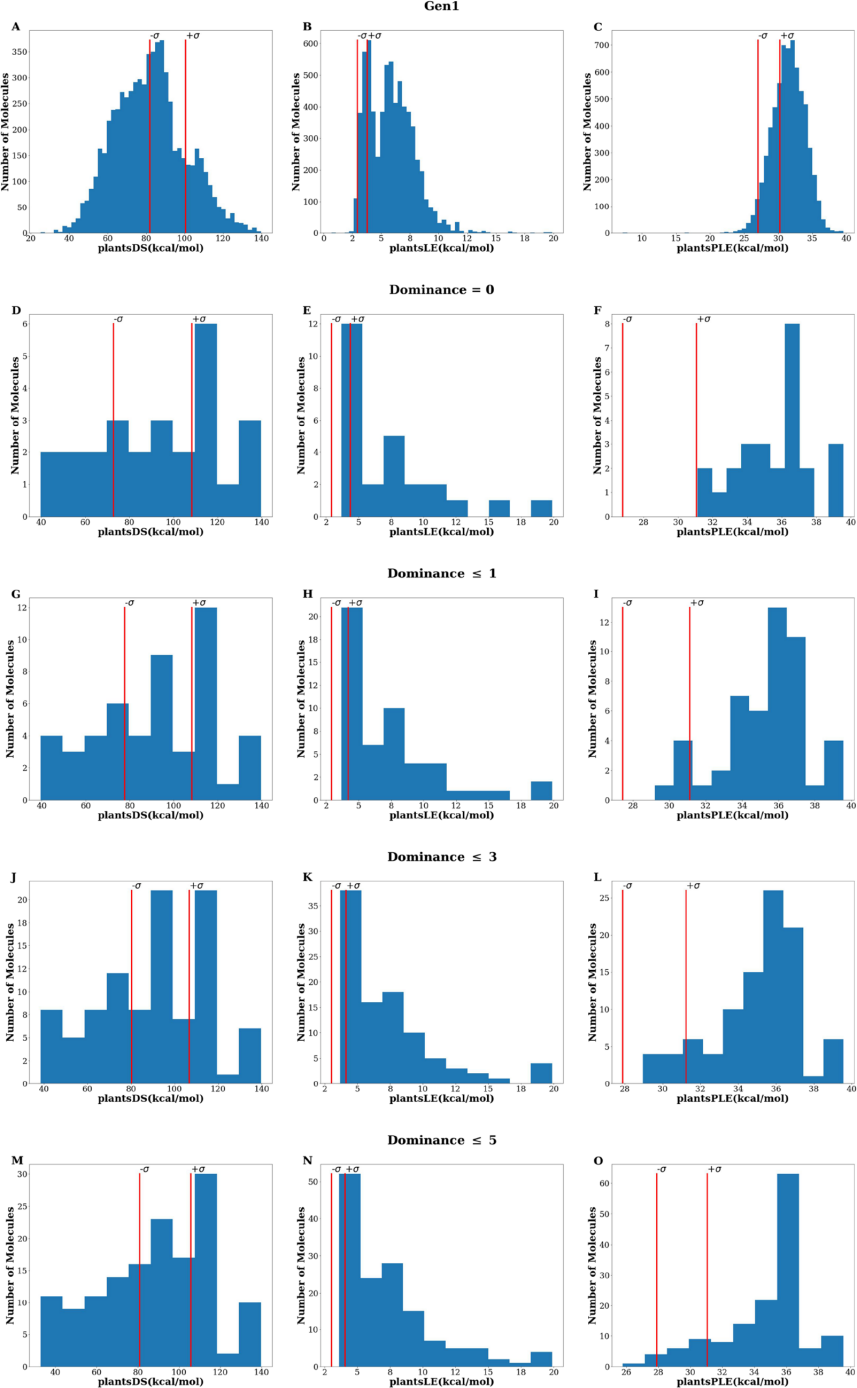
PLANTS^25^ score (plantsDS, plantsLE, and plantsPLE) distributions returned by the *Gen1* set. Red vertical lines indicate the standard deviation limits related to the corresponding data of the reference set. Note: active “seed” compounds defining the (−σ, +σ) range are not counted here.

**Figure 4:**
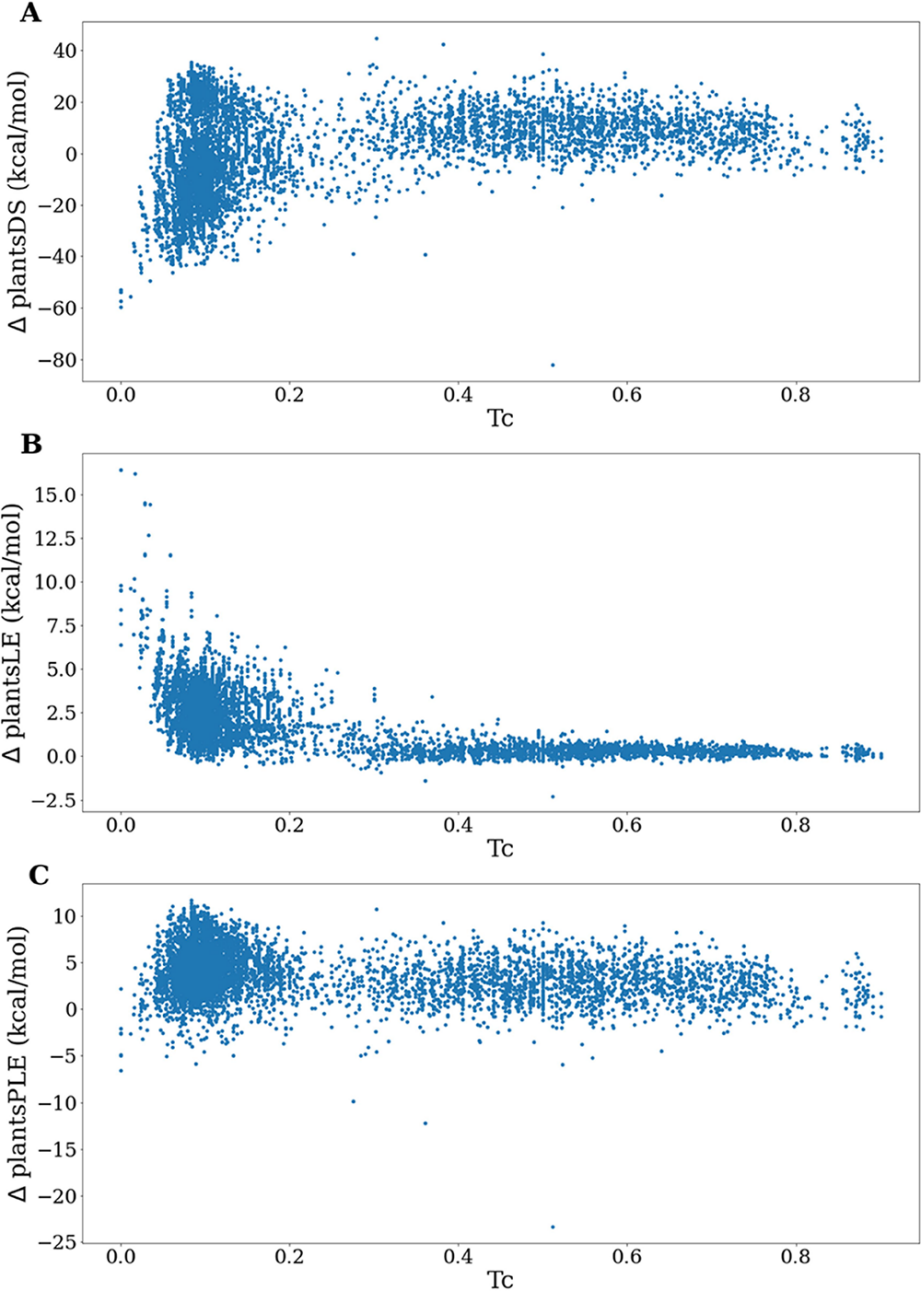
2D graphs obtained by plotting, for each molecule belonging to the *Gen1* set, the similarity (*T*_c_) to the most similar compound of the reference set against (A) ΔplantsDS, (B) ΔplantsLE, and (C) ΔplantsPLE.

**Figure 5:**
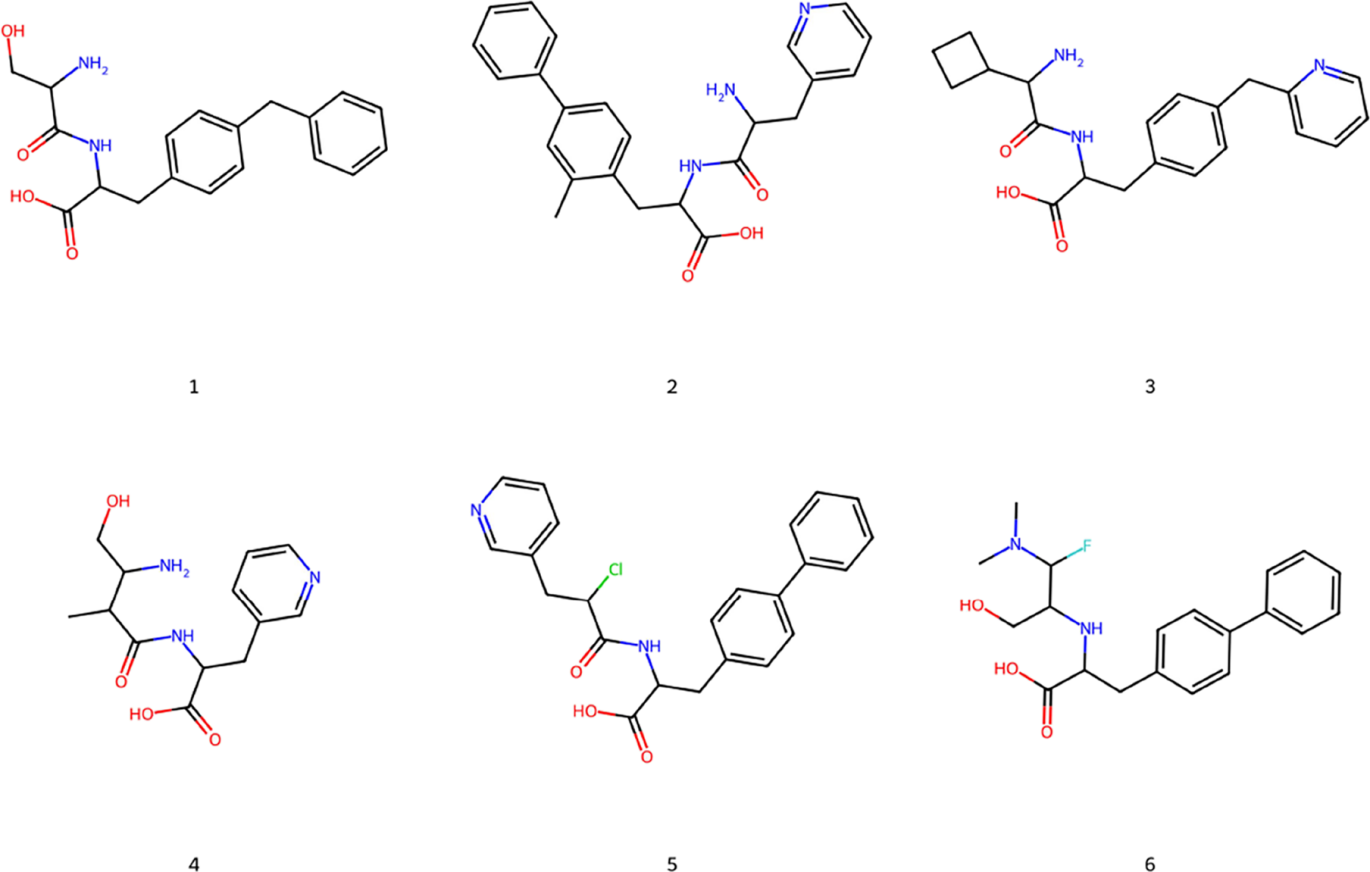
Two-dimensional (2D) structures of some compounds belonging to the *Gen1* set.

Figure 3 shows the PLANTS^25^ score distributions (*plantsDS, plantsLE,* and *plantsPLE*) returned by the generated compounds. An initial examination of the entire *Gen1 set* provides evidence of GENERA’s ability to generate docking compounds correctly. This capability is particularly evident when observing Figure 3C, which demonstrates that most generated compounds exhibit better plantsPLE values than the initial reference set. However, as GENERA was designed to create high-quality, focused libraries from which the best candidates could be selected for in vitro testing, we directed our attention toward the top-performing subsets that are also the same compounds that the algorithm itself rates as “best fitting” given the current objectives. Figure 3D–O displays the docking score distributions computed for the Pareto front of increasing depth, for example, solutions of increasing dominance (*Dom* ≤ 0, 1, 3, and 5). It is worth noting that a significant improvement in plantsPLE is observed in a considerable portion of the generated compounds, regardless of the selected *Dom* threshold, when compared to the reference set. Furthermore, the observed enhancement in plantsLE can be attributed to fragments within the *Gen1 set*, as evident from Figure 2A. Potentially, these fragments can serve as a valuable library for fragment-based VS approaches. Additionally, the quality of the generated set was evaluated by calculating two commonly used metrics in de novo design:^41^ internal diversity^12^ (ID) and synthetic accessibility (SA) score. ID represents the average Soergel^42^ distance (Sc-based on the Morgan fingerprint with radius 2) between each molecule and the others within the same set,^43,44^ while the SA score, introduced by Ertl and Schuffenhauer^45^ ranges from 1 (easy synthesis) to 10 (challenging synthesis). Remarkably, *Gen1 set* exhibited a high ID value of 0.88, confirming the GENERA’s ability to propose diverse molecules. Notably, even considering small subsets consisting only of low-dominated solutions, the ID remained high at 0.88, 0.87, 0.87, and 0.87 for *Dom* thresholds of 0, 1, 3, and 5, respectively. It is worth mentioning that the number of compounds in these subsets is 26, 50, 97, and 143, respectively. In comparison, the active compounds (42) belonging to the reference set are more similar to each other (ID = 0.56). Interestingly, although SA was not used as an objective during the generation, the generated library exhibits an average SA score comparable, if not better than the reference set (3.39 ± 0.75 vs 3.64 ± 0.47). Notably, the small subsets consisting only of low-dominated solutions also returned fair SA values. Indeed, values as low as 3.35 ± 0.79 (*Dom* = 0), 3.32 ± 0.71 (*Dom* ≤ 1), 3.29 ± 0.71 (*Dom* ≤ 3), and 3.31 ± 0.71 (*Dom* ≤ 5) were computed. This finding confirms the ability of the utilized mutational operator (*DeLA-Drug*) to generate analogues with good SA, as previously reported.^24^ Finally, to assess the ability of GENERA to design molecules exploring a new chemical space, we plotted, for each generated molecule, the Tanimoto similarity (*T*_c_-based on the Morgan fingerprint with radius 2^43,44^) to the most similar compound belonging to the reference set against ΔplantsDS (Figure 4A), ΔplantsLE (Figure 4B), and ΔplantsPLE (Figure 4C), computed as follows:
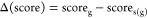
1where **g** represents a generated molecule, while **s(g)** represents **g**’s nearest neighbor compound from the reference set. Notably, most of the generated molecules exhibited *T*_c_ values below 0.6, further supporting GENERA’s ability to design analogues and candidates exploring a new chemical space, a crucial aspect in de novo design.^46^ Moreover, as observed in Figure 4, there is no correlation between the similarity to the reference set and the difference in docking scores, proving that the algorithm can work on areas of the chemical space farther from the starting set and still improve the desired objectives. Indeed, many compounds responsible for the top plantsPLE values return very low Tc. A representative set of the compounds belonging to *Gen1* is depicted in Figure 5. Notice that all the selected molecules display an acidic function, known to be important for the activity toward ACE2 being able to coordinate the zinc ion within the binding site.

### Generation Based on both PLANTS and GLIDE Docking Scores

It is well known that selecting candidates based on molecular docking simulations only has many limitations as the scoring function accuracy is strongly dependent on the specific target being studied.^47^ These weaknesses can be, at least partially, mitigated by combining multiple software tools. Indeed, this approach allows increasing the hit rates of VS campaigns, as reported in previous studies.^48^ Building on this evidence, we challenged GENERA to combine PLANTS^25^ and GLIDE,^26^ both used as inputs for the Pareto front. Specifically, we employed EVS, plantsDS, plantsLE, glideDS, and glideLE as objectives during the generation process. We used the same reference set of active compounds as before and generated a new set of **8336** unique and chemically valid molecules (from now on referred to as the *Gen2* set). Notice that this generation required about 10 days on one CPU only. It is worth noting that *Gen2* consists, on average, of heavier compounds. Indeed, 56% of the compounds belonging to *Gen2* (vs 43% in *Gen1*) have more than 15 heavy atoms. This difference is also evident after comparing the distributions of heavy atoms returned by *Gen1* and *Gen2* sets (Figure 2A vs 2B). This can be explained by the fact that, during the second generation, two out of the five objectives (i.e., *plantsDS* and *glideDS*) drive the generation toward bulkier molecules, compared to only one out of four when only PLANTS^25^ data are considered.

**Figure 6:**
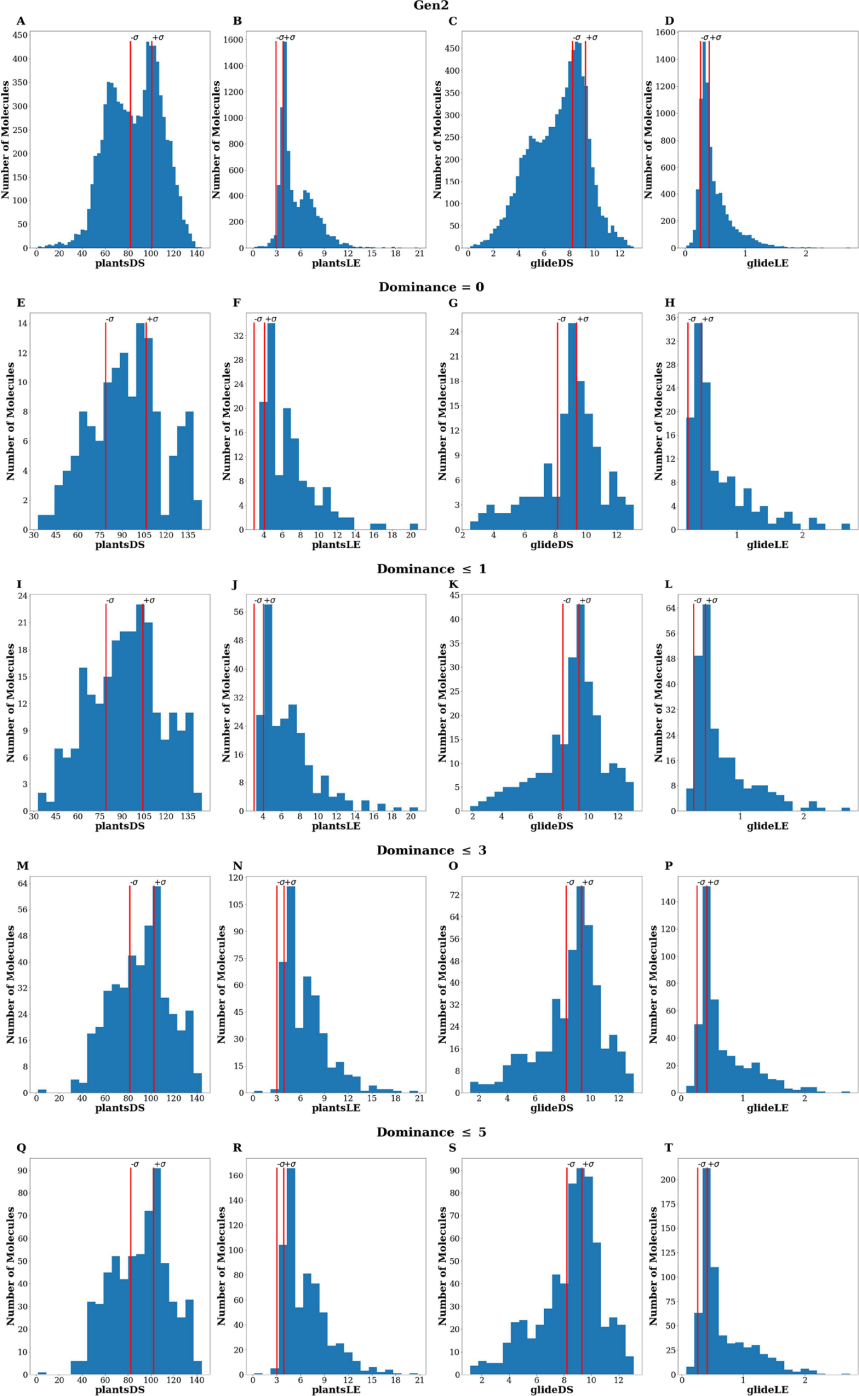
PLANTS^25^ and GLIDE^26^ scores *(plantsDS*, *plantsLE*, *glideDS*, and *glideLE*) distributions returned by the *Gen2 set*. The red vertical lines indicate the standard deviation limits related to the data obtained from the reference set. Note: active “seed” compounds defining the (−σ, +σ) range are not counted here.

**Figure 7:**
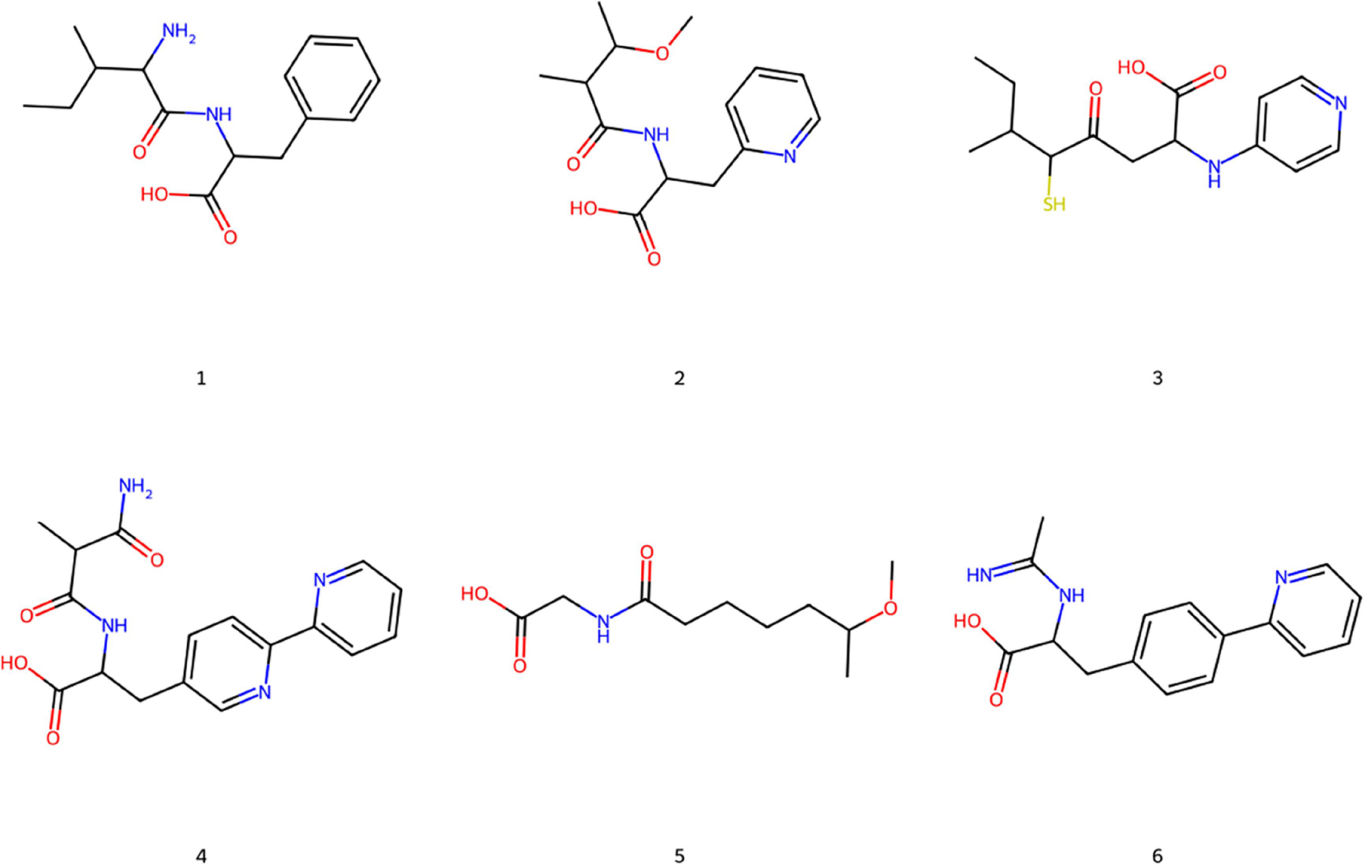
Two-dimensional structures of some compounds generated by *GENERA* and belonging to the *Gen2 set*.

**Figure 8:**
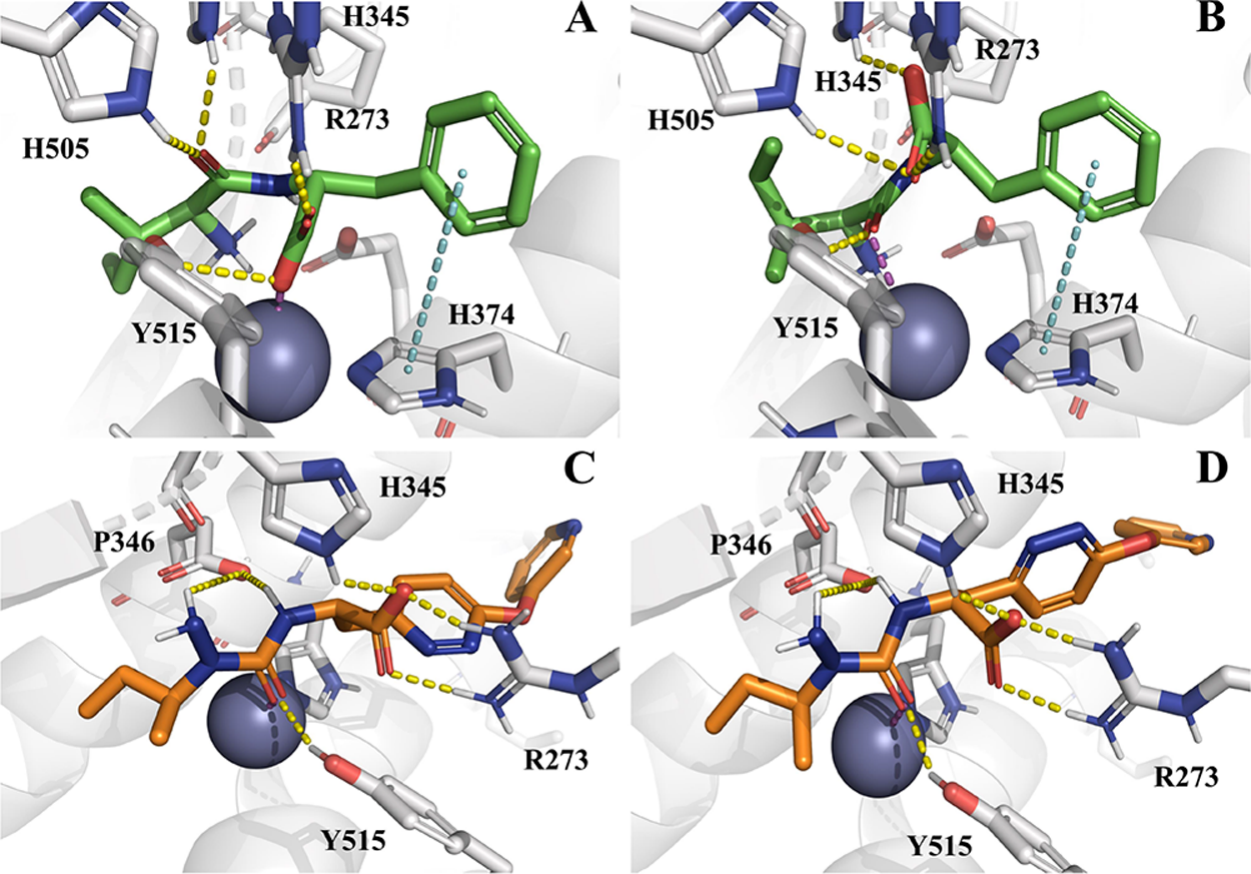
Top-scored docking poses of two compounds belonging to the *Gen2 set*: (A) compound **1** (Figure 7) docked by GLIDE;^26^ (B) compound **1** docked by PLANTS;^25^ (C) compound **6** (Figure 7) docked by GLIDE;^26^ and (D) compound **6** docked by PLANTS.^25^ Ligands and important residues are rendered as sticks, while the protein as cartoon. H-bond, salt-bridge, and ligand-Zn interactions are depicted by a dotted yellow, cyan, and magenta line, respectively. For the sake of clarity, only polar hydrogen atoms are shown. Notice that the blue ball represents the zinc atom within the ACE-2 binding site.

Remarkably, GENERA could design compounds predicted to be even better than the starting active molecules by both PLANTS^25^ and GLIDE.^26^ This is particularly evident when examining the subsets selected based on the computed *Dom* (Figure 6). For example, most of the compounds with a *Dom* value ≤3 (Figures 6M–P) exhibited improvements in both plantsLE and glideLE compared to the reference set. Importantly, even when employing a higher *Dom* threshold for subset selection, the overall quality of the obtained PLANTS^25^ and GLIDE^26^ scores is not significantly compromised, as indicated by the distributions of all the compounds (628) having *Dom* values ≤5. The subsets with a dominance value of 0 (consisting of 135 molecules—ID = 0.86), ≤1 (234 molecules—ID = 0.86), ≤3 (440 molecules—ID = 0.86), and ≤ 5 (628 molecules—ID = 0.87) all demonstrate substantial chemical diversity, exploring different regions of the chemical space. Furthermore, the capability of GENERA to generate compounds with favorable SA, despite the absence of this parameter as a GA objective, is once again confirmed. Average SA values below 3.90 were obtained for *Gen2* and for all the considered subsets comprising compounds with low dominance (using *Dom* = 0, 1, 3, and 5 as thresholds). Moreover, it is interesting to note that, as seen in *Gen1*, a significant number of the generated compounds have low similarity with the reference set (Figure S1 in the Supporting Information) but still show improved scores. This further supports the potential of GENERA to design novel compounds that explore unexplored regions of the chemical space while maintaining good target-related properties used as objectives. A representative set of the compounds belonging to the *Gen2* set is shown in Figure 7 while Figure 8 shows the top-scored docking poses returned by two of them. It is noteworthy that GENERA was re-tested using a pool of 10 compounds known to be inactive toward ACE2 (CHEMBL261033, CHEMBL405913, CHEMBL258464, CHEMBL405232, CHEMBL258683, CHEMBL412123, CHEMBL264665, CHEMBL409713, CHEMBL404044, and CHEMBL163454) as the starting set. Among the generated set of 1500 molecules, we generated a significant number of molecules returning plantsDS (131 compounds), plantsLE (437), glideDS (422), and glideLE (239) better than the top plantsDS (110.6 kcal/mol), plantsLE (4.80), glideDS (9.5 kcal/mol), and glideLE (0.58) displayed by the reference set of active molecules.

### GA Impact on the GENERA Workflow

**Figure 9:**
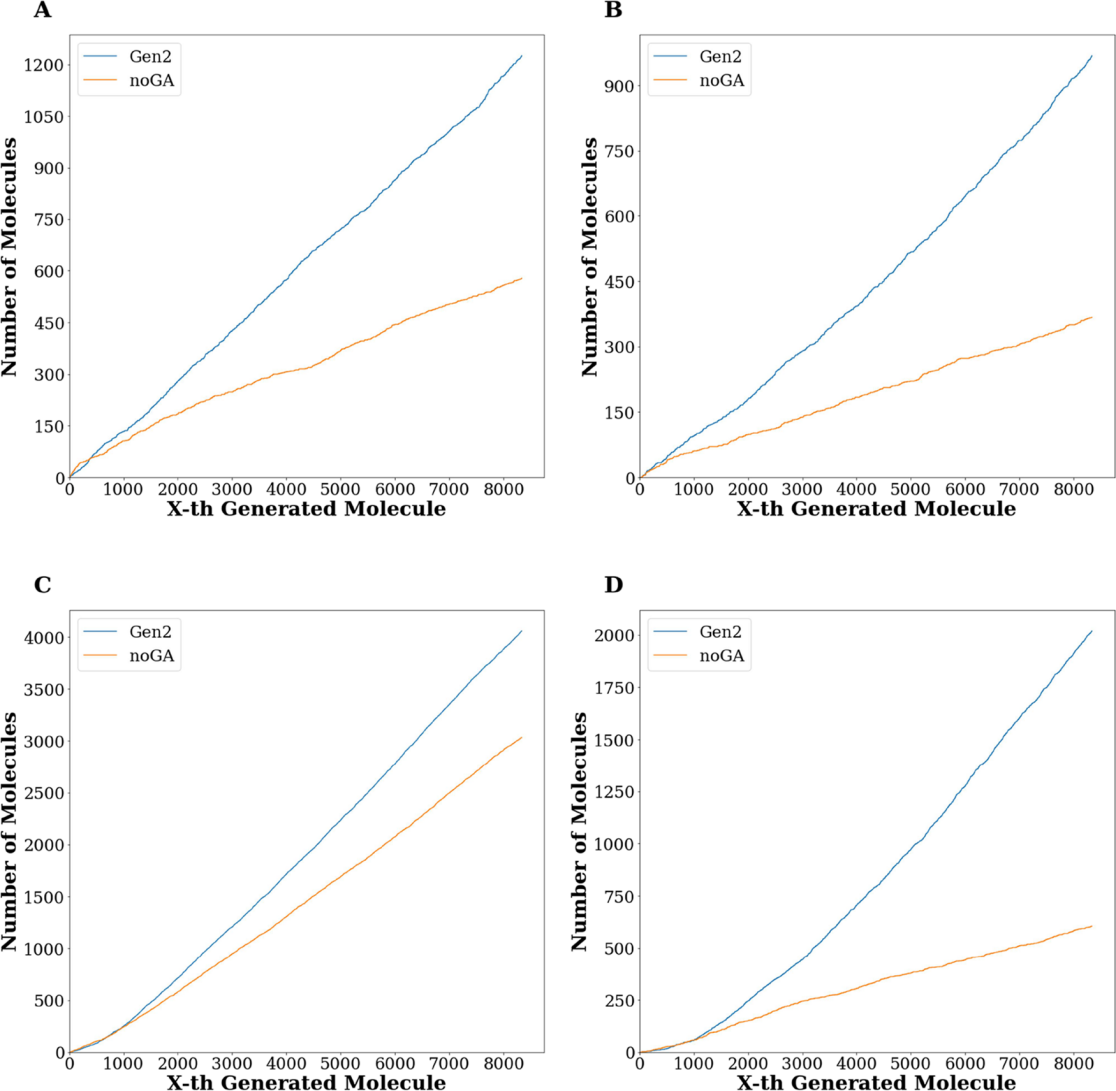
Two-dimensional graphs obtaining by plotting the number of compounds belonging to the *Gen2 set* (blue line) and *noGA set* (orange line) returning (A) plantsDSs better than the top plantsDS displayed by the reference set (110.6 kcal/mol), (B) plantsLE better than the top plantsLE displayed by the reference set (4.80), (C) glideDSs better than the top glideDS displayed by the reference set (9.5 kcal/mol), and (D) glideLE better than the top glideLE displayed by the reference set (0.58).

Aimed at further weighing the ability of *GENERA* to perform differently from *DeLA-Drug* taken alone, a target-oriented automated design, a new generation, was performed. The only modification made to the GENERA architecture presented in Figure 1 was the selection of the best population based on random sampling rather than a docking-based fitness function. In other words, we re-used the architecture of *GENERA* without using its GA. This set of generated compounds was compared to the previously obtained *Gen2*. In particular, for the sake of comparison (i.e., equally-sized datasets), **8336** unique and chemically valid molecules were generated by *DeLA-Drug* alone, using again a number of substitutions equal to 5 (hereinafter referred to as *Gen2_NoGA*). Figure 9 shows 2D plots reporting the time dependence (based on the generation order) of the number of compounds belonging to the *Gen2* set (blue line) and *Gen2_NoGA* (orange line) returning (A) plantsDSs better than the top plantsDS displayed by the reference set, (B) plantsLE better than the top plantsLE displayed by the reference set, (C) glideDSs better than the top glideDS displayed by the reference set, and (D) glideLE better than the top glideLE displayed by the reference set. As evident from Figure 9, the employed GA is crucial in improving the generated compounds in terms of the docking metrics employed to build the fitness function (i.e., DSs and LEs returned by both PLANTS^25^ and GLIDE^26^).

**Figure 10:**
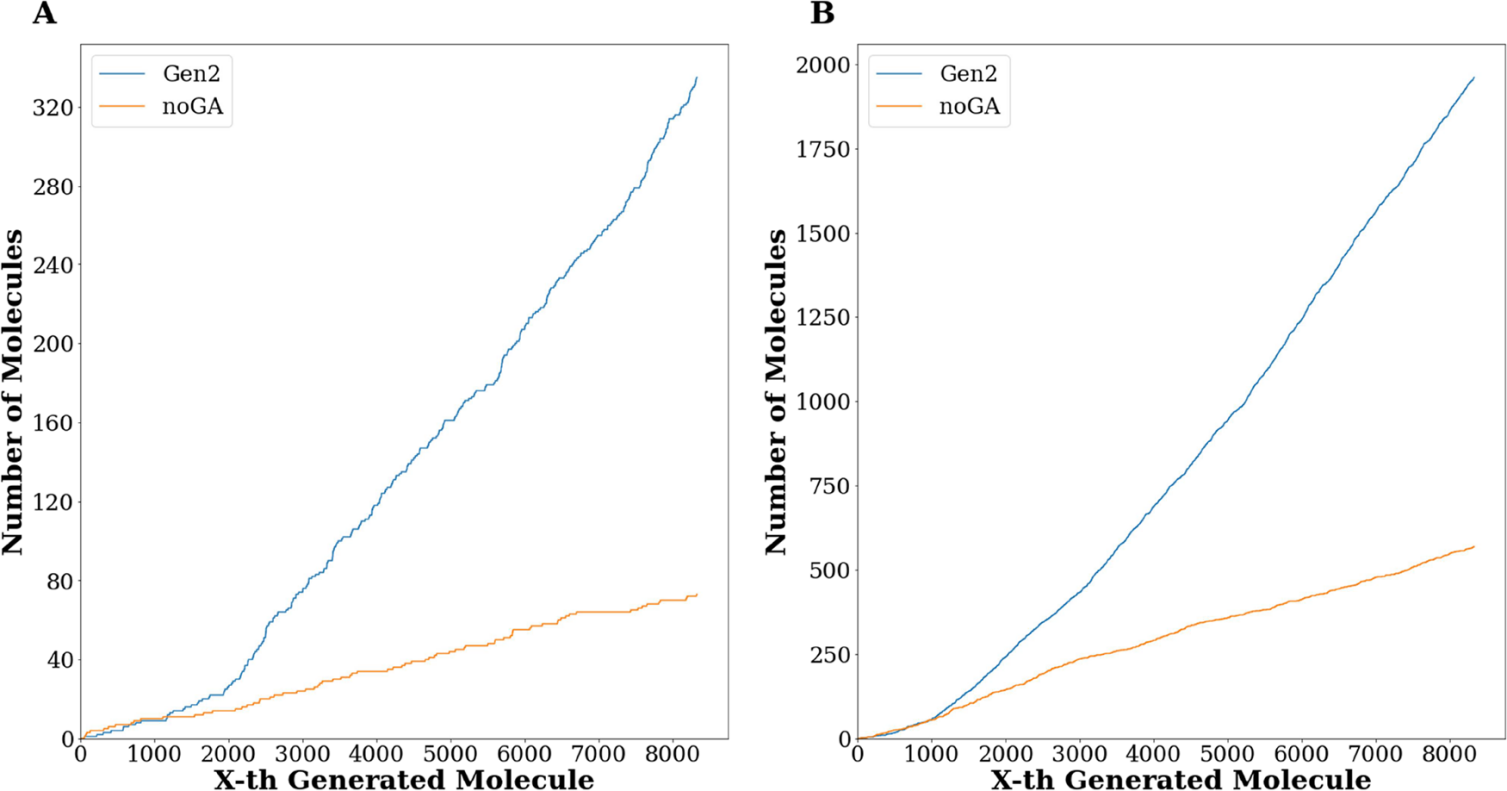
Two-dimensional graphs obtained by plotting the number of compounds belonging to the *Gen2 set* (blue line) and *noGA set* (orange line) returning at the same time (A) PLANTS^25^ DSs better than the top DS displayed by the *parents set* (110.6 kcal/mol) and GLIDE^26^ DSs better than the top DS displayed by the parents set (9.5 kcal/mol); (B) PLANTS^25^ LEs better than the top LE displayed by the reference set (4.80) and glideLEs better than the top LE displayed by the reference set (0.58).

Finally, the ability of GENERA to perform a multiobjective optimization was further assessed by plotting the time dependence of the compounds matching two criteria simultaneously. The criteria were coupled based on whether or not the objectives are scaled based on the molecule’s size. Thus, Figure 10A shows, at each time step, how many molecules have both plantsDS and glideDS better than the best respective objectives in the reference set, while Figure 10B is analogous but considers plantsLE and glideLE simultaneously. GENERA designed a higher number of compounds matching these criteria compared to *DeLA-Drug* employed alone (335 vs 73 and 1961 vs 568 for DSs and LEs, respectively). This again put forward GENERA as a valid tool for multiobjective de novo design.

## Conclusions

Our study introduces *GENERA*, a novel algorithm that combines *DeLA-Drug*, a recurrent neural network model for analogue generation, with a genetic algorithm framework. The analysis of the focused libraries generated by *GENERA* revealed its ability to quickly optimize user-defined properties. Deep-learning-powered tools which can make random but nonetheless chemically valid steps in chemical space are, as could be shown here, one of the most valuable contributions of the deep-learning field in chemistry. Indeed, the real bottleneck in random walking the chemical space is the ability to define “mutations” leading with a high probability to valid chemical structures—which is prohibitive when trying to operate directly on SMILES. Even compared to custom-made molecular representations aimed at being “easy” to modify,^49^ the use of *DeLA-Drug* has clear advantages. First, it can be implemented “out-of-box” into any evolutionary algorithm template without conceiving any data structure-specific mutation procedures. Foremost, using *DeLA-Drug* implicitly lets the user benefit from the “chemistry knowledge” learned from ChEMBL during its training. Albeit not perfect, resulting structures were most often chemically meaningful, could be processed by ligand preparation tools, assigned force field parameters, and docked—which is the first, robust indicator of chemical validity. Last but not least, focusing on deep-learned “chemistry knowledge” to power the sampling of chemical space specifically has the important merit of flexibility because it does not impose any prerequisites in terms of usage. Because the objective functions can be seamlessly coupled to the tool—any executable or script accepting SMILES as the input and returning a goodness score as the output will do—the method supports (multi)objective optimization of endpoints of any nature. By contrast, including affinity data in the deep-learning process for de novo focused library design only makes sense for targets with a wealth of structure–activity data already harvested (hence probably no longer of interest for drug designers). With a generic, robust “mutation operator”, the evolutionary process is more than able to attract structures toward targeted chemical space zones, irrespective of whether these chemical space zones are defined as a Tanimoto similarity sphere around a reference active in a 2D fingerprint space or an estimated free energy from (however sophisticated) docking. We demonstrated the efficacy of the algorithm by applying it to the design of ACE2 inhibitors, producing compounds with optimized target-dependent properties based on docking scores from PLANTS^25^ and GLIDE^26^ programs. *GENERA* proved to be a valuable tool for multiobjective optimization, as the generated focused libraries outperformed the starting reference library of ACE2 active compounds with regard to the objectives used during the generation. In summary, our algorithm quickly designed compounds (i) predicted to be even more affine toward the target than those in the starting reference set of known ACE-2 inhibitors, (ii) with good SA, which represents the main concern in de novo design projects,^50^ and (iii) exploring a new chemical space. These results highlight GENERA’s potential as an innovative computational workflow for target-oriented de novo design, offering the flexibility to optimize, starting from a reference pool of compounds, relevant properties such as the predicted target affinity, drug-likeness, or any user-defined target-related property.

## Data Availability

GENERA is freely available in a GitHub repository (https://github.com/GiuseppeLamanna/GENERA).

## Supporting Information Available

2D graphs obtained by plotting, for each molecule belonging to the Gen2 set, the similarity (*T*_c_) to the most similar compound of the reference set against ΔplantsDS, ΔplantsLE, and ΔplantsPLE (PDF)
