# Supplementary material for: GENERA: A Combined Genetic/Deep-Learning Algorithm for Multiobjective Target-Oriented De Novo Design
Source: J Chem Inf Model. 2023 Aug 9;63(16):5107–19. doi: 10.1021/acs.jcim.3c00963 (PMC10466378; doi:10.1021/acs.jcim.3c00963)
Supplement: Supplementary file 1 — ci3c00963_si_001.pdf [file ci3c00963_si_001.pdf]

Supporting Information

GENERA: a combined GENEtic/deep leaRning Algorithm for multi-objective target-oriented de-novo design

Giuseppe Lamanna[a,b], *Pietro Delre,*[b] *Gilles Marcou,*[c] *Michele Saviano,*[d] *Alexandre Varnek,*[c]

Dragos Horvath[c]* *and Giuseppe Felice Mangiatordi*[b]*

[a] Chemistry Department, University of Bari "Aldo Moro", via E. Orabona, 4, I-70125 Bari, Italy.

[b] CNR – Institute of Crystallography, Via Amendola 122/o, 70126 Bari, Italy

[c] Laboratoire de Chémoinformatique UMR7140, 4 rue Blaise Pascal, 67000 Strasbourg, France

[d] CNR – Institute of Crystallography, Via Vivaldi 43, 81100, Caserta, Italy

*Correspondence: giuseppe.mangiatordi@ic.cnr.it, dhorvath@unistra.fr

Content

Figure S1. 2D graphs obtained by plotting, for each molecule belonging to the *Gen2 set*, the similarity (Tc) to the most similar compound of the *reference set* against A) -ΔplantsDS, B) -ΔplantsLE and C) - ΔplantsPLE.

Figure S1.

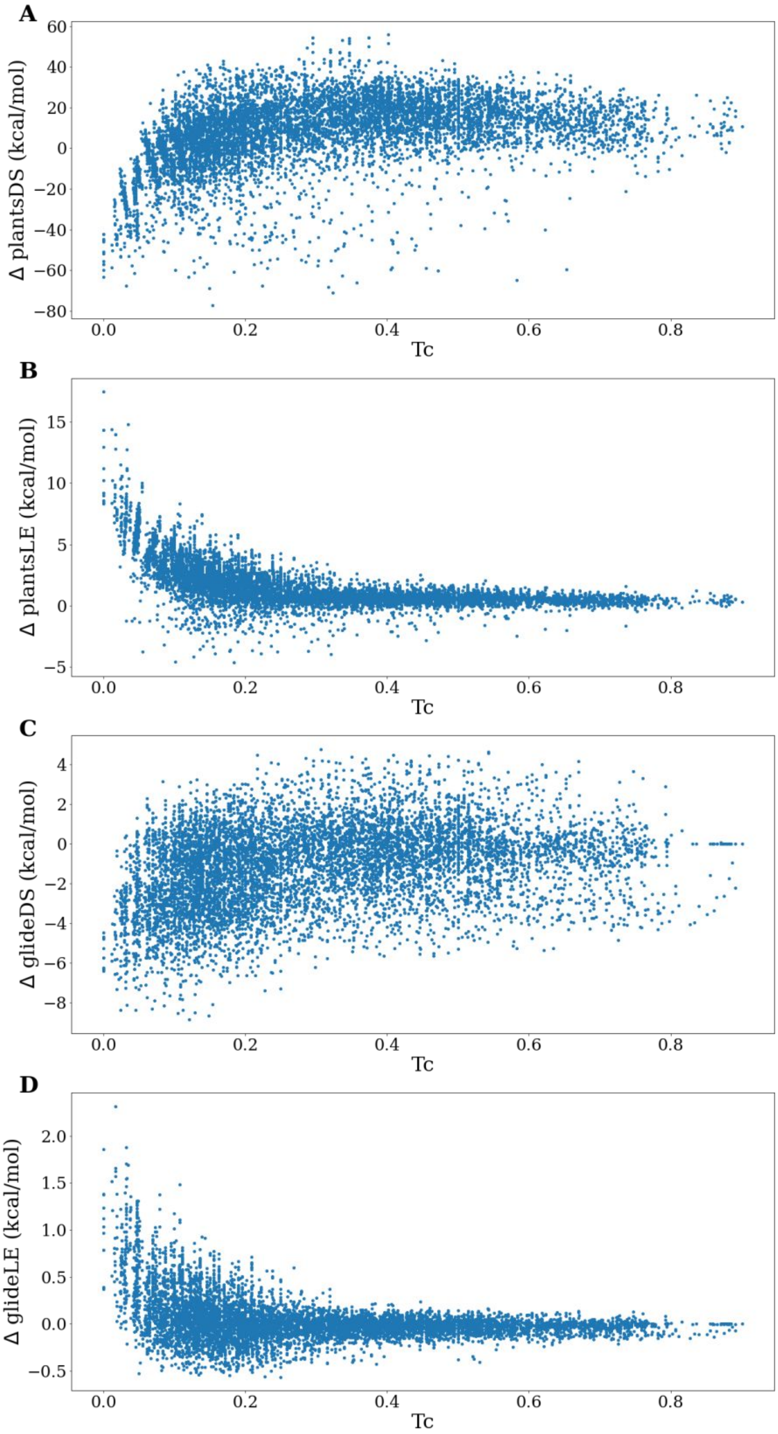

A
B
C
D
Δ plantsDS (kcal/mol)
Δ plantsLE (kcal/mol)
Δ glideDS (kcal/mol)
Δ glideLE (kcal/mol)
Tc
